# Wildfire risk for main vegetation units in a biodiversity hotspot: modeling approach in New Caledonia, South Pacific

**DOI:** 10.1002/ece3.1317

**Published:** 2014-12-28

**Authors:** Céline Gomez, Morgan Mangeas, Thomas Curt, Thomas Ibanez, Jérôme Munzinger, Pascal Dumas, André Jérémy, Marc Despinoy, Christelle Hély

**Affiliations:** 1 I.R.D. UMR ESPACE DEVBP A5, 98848, Nouméa Cedex, New Caledonia; 2 Irstea, UR EMAX Ecosystèmes Méditerranéens et Risques13182 Aix en Provence, Cedex 05, France; 3 Diversités biologique et Fonctionnelle des Écosystèmes Terrestres, Institut Agronomique néo-Calédonien (IAC)BP A5, 98800, Nouméa, New Caledonia; 4 UMR AMAP, Laboratoire de Botanique et d'Ecologie Végétale Appliquées, Herbarium NOU, Centre IRDBPA5, 98848, Nouméa, New Caledonia; 5 IRD, UMR AMAP34398, Montpellier, France; 6 EA 4242 Centre for New Pacific Studies (CNEP), University of New CaledoniaNouméa, New Caledonia; 7 Centre de Bio-Archéologie et Ecologie, UMR 5059, EPHE (Laboratoire PALECO)34090, Montpellier, France

**Keywords:** Biodiversity loss, burn probability, fire impact, FLAMMAP, NEW Caledonia, spatially explicit modeling, wildfire risk assessment

## Abstract

Wildfire has been recognized as one of the most ubiquitous disturbance agents to impact on natural environments. In this study, our main objective was to propose a modeling approach to investigate the potential impact of wildfire on biodiversity. The method is illustrated with an application example in New Caledonia where conservation and sustainable biodiversity management represent an important challenge. Firstly, a biodiversity loss index, including the diversity and the vulnerability indexes, was calculated for every vegetation unit in New Caledonia and mapped according to its distribution over the New Caledonian mainland. Then, based on spatially explicit fire behavior simulations (using the FLAMMAP software) and fire ignition probabilities, two original fire risk assessment approaches were proposed: a one-off event model and a multi-event burn probability model. The spatial distribution of fire risk across New Caledonia was similar for both indices with very small localized spots having high risk. The patterns relating to highest risk are all located around the remaining sclerophyll forest fragments and are representing 0.012% of the mainland surface. A small part of maquis and areas adjacent to dense humid forest on ultramafic substrates should also be monitored. Vegetation interfaces between secondary and primary units displayed high risk and should represent priority zones for fire effects mitigation. Low fire ignition probability in anthropogenic-free areas decreases drastically the risk. A one-off event associated risk allowed localizing of the most likely ignition areas with potential for extensive damage. Emergency actions could aim limiting specific fire spread known to have high impact or consist of on targeting high risk areas to limit one-off fire ignitions. Spatially explicit information on burning probability is necessary for setting strategic fire and fuel management planning. Both risk indices provide clues to preserve New Caledonia hot spot of biodiversity facing wildfires.

## Introduction

Wildfire is one of the most ubiquitous terrestrial disturbance (Bowman et al. 2009) and widely impacts natural environments (Hochberg et al. 1994; Kass et al. 2011). These impacts occur on different spatial scales, modify landscape structures (Hochberg et al. 1994), increase habitat fragmentation (Cochrane 2001), and change the species composition of ecosystems (Trabaud 1994; Dìaz-Delgado et al. 2004). Since the beginning of the 1980s, wildfires have emerged as an increasing threat for tropical rainforest (e.g., Nepstad et al. 1999; Siegert et al. 2001; Cochrane 2003). Nevertheless, despite important stakes for biodiversity, the ecology of fire in Tropical ecosystems remains poorly known compared to the ecology of fire in Mediterranean or Boreal ecosystems. Developing tools at large scales is currently needed to prevent wildfire impacts and represents a strategic stake in tropical ecosystems for the conservation of biodiversity.

Indeed, tropical rainforests have a low flammability (Cochrane 2003) but are particularly impacted by wildfires since tropical rainforest species are poorly adapted to fire (Nepstad et al. 1999; Barlow and Peres 2008; Carvalho et al. 2010; Granzow-de la Cerda et al. 2012). Although few data are available in tropical rainforests (but see Balch et al. 2011; Brando et al. 2012), trees commonly exhibit high postfire mortality rates in these ecosystems (about 40% for large DBH >= 10 cm trees, Barlow and Peres 2008). This high tree postfire mortality open and fragment these ecosystems favoring the emergence of new fires (Nepstad et al. 1999; Carvalho et al. 2010; Granzow-de la Cerda et al. 2012). Moreover, the recurrence of such fires commonly leads to the conversion of forests in open fire-prone ecosystems such as savannahs (Barlow and Peres 2008).

Fire ignition causes are multiple but in most countries fires are ignited accidentally or deliberately by man in the frame of different activities such as heat, cooking, hunting, and above all land clearing (invasive species control or agriculture). More than 90% of fires, in the Mediterranean region, are man-made (Chuvieco et al. 2009) and man is by far the main source of fire ignitions in the tropics (Stott 2000).

The impact of wildfire is a function of many parameters acting on different spatio-temporal scales (Perry et al. 1999; Syphard et al. 2008; Hayes and Robeson 2011). Potential impacts at small spatio-temporal scales depend on fire behaviors (i.e., surface or crown fire, flame front depth, intensity, and rate of spread or flame length), while impacts at large spatio-temporal scales depend on fire regimes (e.g., frequency of fires, size, season or intensity). Fire frequency and spatial structure are largely influenced by changes to the landscape patterns and the risk generating process (ignition and fire spread) (Massada et al. 2009). For instance, shift from low to high frequency fire regimes could be dramatic by converting landscapes initially composed of nonflammable stages into highly flammable landscapes supporting large and frequent fires (Kitzberger et al. 2012). Two levels of fire impact can thus be distinguished: (1) the impact of a specific fire event relating to the ignition location, the burnt area and the varying fire behavior within it, and (2) the impact on a specific geographic location which experiences multiple wildfires constrained by the structural landscape and climatic configuration.

Experimental burns provide useful insights about tree mortality (e.g., Balch et al. 2011) that may be introduced in global fire risk assessment framework. However, such data are difficult to collect, and fire modeling remains of particular importance for understanding fire dynamics. Assumptions are needed to simulate fire spread and its physical components (fire spread rate, heat release, and flame size) from driving variables such as the relationship between fuel structure and fire spread (Keeley 2002; Williams et al. 2002; Pinol et al. 2007). Fire depends on physical, biological, and/or ecological components, and this leads to complex fire behavior and thus to multiple potential effects (Cochrane 2003). To some extent, the factors that permit ignition will also influence fire behavior. For instance, well-aerated, fine fuels will burn more intensely and spread more rapidly in addition to being more likely to ignite (Whelan 1995).

Risk assessment terminology has long been confusing, talking about fire danger or fire hazard without providing clear definitions. Bachmann and Allgower (2001) and later, Hardy (2005) specified the definition of risks, hazards, and severity of wildfire. Wildfire risk has to be considered as the probability of a wildfire occurring at a specified location and under specific conditions, together with its expected outcome as defined by its impact on the objects it affects (Bachmann and Allgower 2001). The burn probabilities defined as a spatially explicit pattern of putative wildfires occurrences on a specific landscape (Miller et al. 2008) and the estimation of intensity and spread rates introduced in (Finney 2005) provide an adequate formalism for estimating this risk. More recently, Chuvieco et al. (2010) proposed a fire risk scheme considering fire ignition, propagation probabilities, and potential damage. Thus, this scheme for risk assessment was designed to be a product of fire danger (ignition and propagation) and potential damages. Within this approach, the behavior and the impact of each individual fire are taken into account.

Based on these previous studies, we developed a novel modeling approach to investigate the potential impact of wildfire on biodiversity. The method is illustrated with an application example in New Caledonia. In this archipelago, located in the South-West Pacific, wildfires combined to mining activities and logging has led to the retreat of natural forests (humid forest and dry forest) to the benefit of anthropogenic secondary open vegetation such as shrublands, thickets, or savannas. Two levels of fire risk have been considered and two fire risk indices were developed as follows: (1) the “One-off event” risk index, which represents the risk associated with a specific fire event, ignited on a given location (i.e., if a fire starts in a specific location, what is the associated risk?), and (2) the “Multi-event” risk index, which represents the risk associated with several fire events, ignited in several locations (also known as a burn probability risk).

The overall objectives of this study were to provide the first wildfire risk assessment modeling at the territory scale to estimate the potential ecological impact of wildfire on the different types of vegetation in New Caledonia, to assess the risk on vegetal biodiversity and finally to provide recommendations to environmental fire management agencies. To reach these objectives, we present a complete process, from biodiversity index definitions, fire ignition and fire risk models combination with field and expert data as input, up to the biodiversity impact analysis. We address also a number of methodological issues such as the necessity to access to high quality geolocalized biodiversity information, the need to consider the human influences within the risk modeling and the interest of providing a one-off event risk and a multi-event risk for different decision support purposes.

## Material and Methods

**Figure 1:**
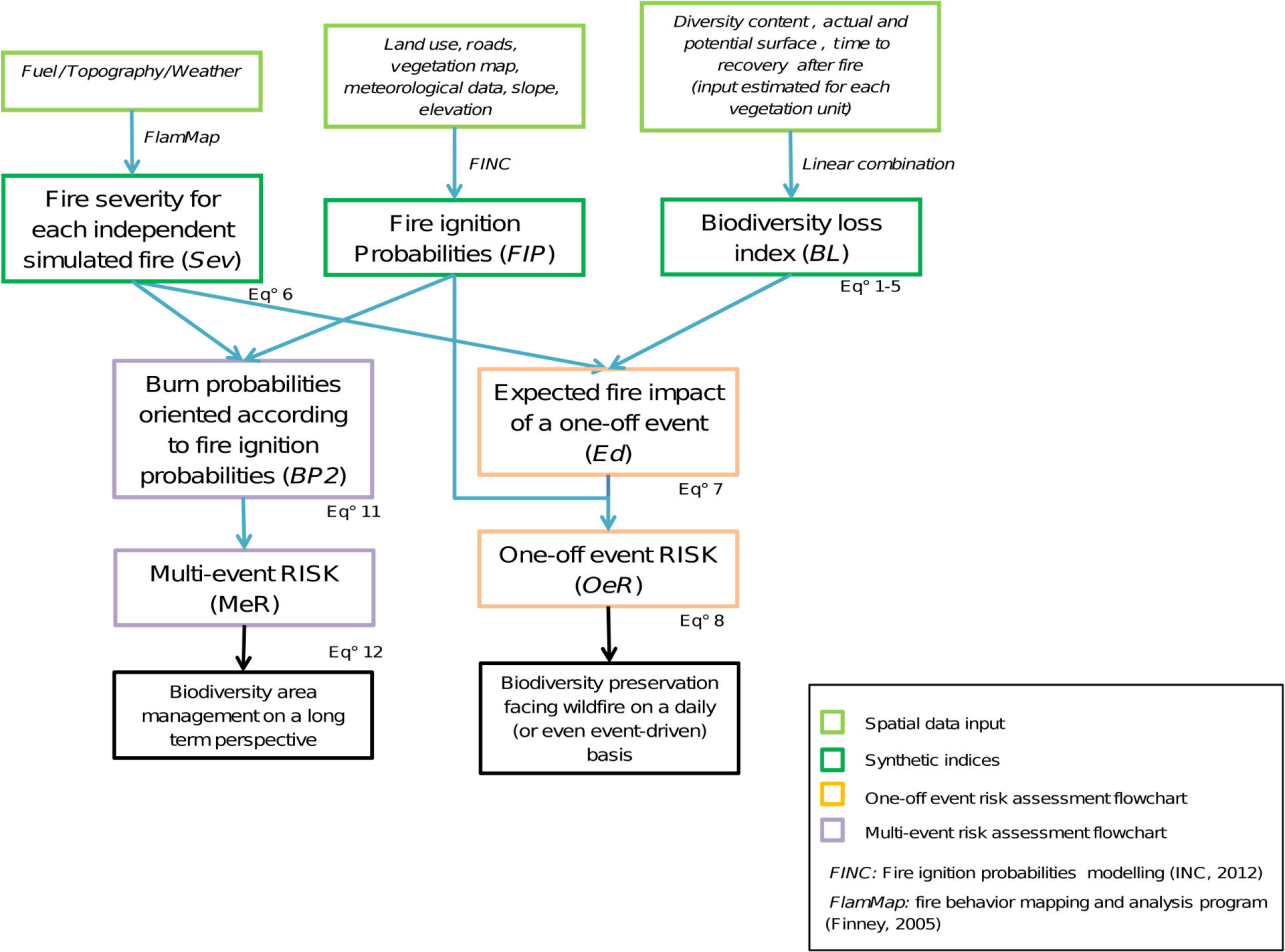
Flowchart of the methodology employed in this study to assess fire risk on vegetal biodiversity through two specific fire risk indices.

A complete flowchart has been designed to help following every step of the methodology referencing each equation, each input, and each output (Fig.1).

### Study site

**Figure 2:**
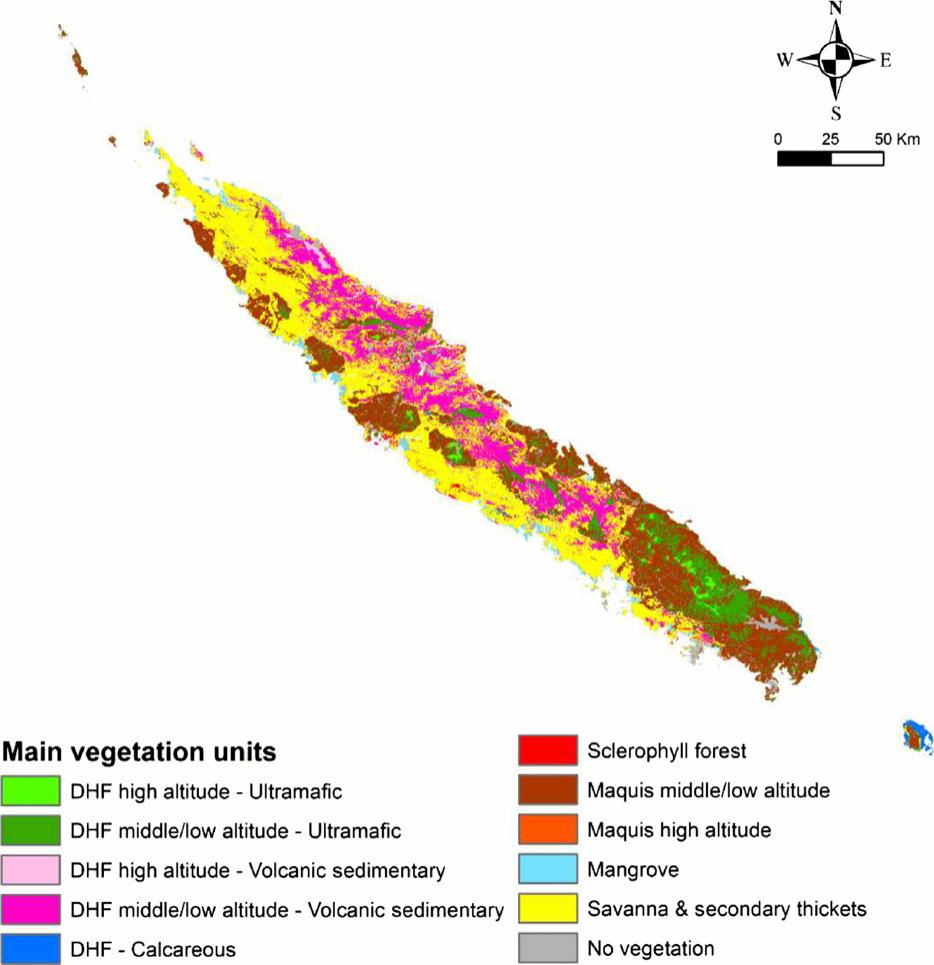
Main vegetation units mapping obtained from land cover, elevation, and soil nature data. No vegetation class included clouds, bared soil, and houses. DHF: Dense Humid Forest; Ultramafic, volcanic-sedimentary and calcareous referred to the soil nature.

New Caledonia (see location on Fig.2) is the smallest terrestrial biodiversity hotspots and recognized as a priority for biodiversity conservation (Myers et al. 2000; Mittermeier et al. 2004). The archipelago encompasses 3371 indigenous vascular plant species, of which 74.7% are endemic (Morat et al. 2012). New Caledonia consequently ranks third in the world for floristic endemism, after Hawaii (89%) and New Zealand (82%). Historic records and charcoal soil profiles, in New Caledonia, show that fire frequency has increased with human settlement (Stevenson 2004). Primary vegetation units (particularly dense humid forests and sclerophyll forests) have regressed in favor of secondary ones (savannas and sclerophyll shrublands, here “maquis minier” and called hereafter maquis (McCoy et al. 1999). Secondary or anthropogenic formations have replaced original vegetation as human settlement and their use of fire (Jaffré et al. 1995; Stott 2000) and are continuously maintained and extended by frequent fires (Jaffré et al. 1998b; Ibanez et al. 2013b).

The recent multidisciplinary INC research project (INC 2012) has been a noteworthy source for information concerning fire issues in New Caledonia, by providing among other outcomes, a historical database of fire ignition and spread, a fuel map and an integrated spatially explicit model of fire ignition risks. To go further and assess the risk of wildfire on vegetal biodiversity, several concepts needed to be defined and integrated such as fire behavior, impact, fire frequency, and risk.

### Vegetation units in New Caledonia

A vegetation map and biodiversity indices per vegetation units were compiled from available published data (Jaffré and Veillon 1994; Jaffré et al. 1997, 1998a, 2009) (See Appendix S1). The main vegetation units (mapped in Fig.2) attributes were grouped in Table1 with potential and actual areas, time to recover after fire (*Time*), total number of species, number of endemic species, number of specific endemic species (i.e., restricted to a specific unit). The spatial resolution was fixed to 300 m × 300 m, which fits in with the intrinsic spatial errors of remote sensing data used for fire ignition modeling development based partly on NDVI burnt area and hotspot MODIS products (Giglio et al. 2009). To preserve the spatial information, as much as possible, from land cover to substrates vector data, we decided to calculate and record the fraction of corresponding classes contained in each cell. This method further allowed cell calculations of biodiversity and vulnerability indices proportional to the percentage of coverage (*ρ*) in each vegetation unit.

**Table 1:** Main vegetation unit attributes used in the present study to compute the biodiversity loss index

Vegetation unit	Potential area (km^2^)	Actual area (km^2^)	Time to recover^2^	Total Species (TS)	Endemic Species (ES)	Specific Endemic Species (SES)	Diversity	Vulnerability	Biodiversity Loss
Dense humid forest	14,745	6570	1	2013^1^	1655^1^	1160^1^	–	–	–
Calcareous substrates	2145	1720	0.5	225^2^	108^2^	23^2^	0.000075	0.6	0.000045
Ultramafic substrates	5891	1770	1	1360^2^	1121^2^	574^2^	0.00072	3.3	0.002376
Sedimentary and volcanic substrates	6709	3080	0.3	1367^2^	1048^2^	481^2^	0.00038	0.65	0.000247
Maquis	480	4267	0.5	1144^1^	1016^1^	534^1^	–	–	
Middle and low altitude	473	4223	0.5	1100^3^	979^3^	21*	0.00022	0.05	0.000011
High altitude	7	44	0.5	200^3^	182^3^	242*	0.0034	0.08	0.000272
Sclerophyll forest	1648	52	0.5	424^1^	233^1^	67^1^	0.0053	15.8	0.08374
Savanna and secondary thickets	0	5311	0.01	410^1^	45^1^	4^1^	0.000027	0	0
TOTAL	–	–	–	3260	2412	1437	–	–	–

1 Jaffré et al. (2009) PSI Tahiti.

2 Jaffré et al. (1997).

3 Jaffré et al. (1998) Threatened plant of NC.

* Values estimated according to botanical expertise establishing that 22% of endemism is generally observed in New Caledonian maquis.

Because of a lack of data about species or diversity distribution and fire ecology in most of the countries and in New Caledonia in particular, as well as the broad spatial scale of our modeling exercise, we assumed that (1) each vegetation unit has a similar composition of species all over the island, and (2) all the species which belong to a vegetation unit are impacted in the same way. Those two assumptions represent a strong simplification of the reality, given the fact that the species are not equally affected (negatively and sometimes positively) by wildfires.

### Computing the biodiversity loss index

General indices were calculated to quantitatively compare the richness, diversity, and vulnerability of previously identified and delimited vegetation units in New Caledonia. Two indices were constructed to characterize units, not only in terms of vegetation diversity and richness, but also in terms of vulnerability to wildfires. Both indices were calculated at the vegetation unit level and finally computed to define a global biodiversity loss (*BL*) index.

The first index described the diversity contents for each vegetation unit (*DVU*) combining three ratios based on richness (i.e., the total number of species, *TS*), number of endemic species (*ES*) and number of specific endemic species (*SES*) recorded within the vegetation units (*u* among the set of vegetation units *U*) and the overall New Caledonia mainland (*L*). These three ratios have linearly been combined and normalized by the actual surface area of the corresponding vegetation unit (*AS*_*u*_):

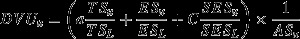
1

Weight coefficients (*a*,*b* and *c*) were set to 1, but can be set differently to meet the biodiversity management requirements(e.g., habitat and/or species conservation).

Based on each vegetation unit's estimated diversity index, the vegetation diversity value contained in any given cell *i* and denoted *D*_*i*_ was calculated. This was performed by summing *DVU*_*u*_ weighted by the cell area (*S*_*i*_) and its composition in vegetation units (*ρ*_*i,u*_) over all the vegetation units within the given cell *i* ∈ *L*, where *L* denotes the set of 300 mx300m cells located on New Caledonia mainland. This index characterizes the diversity content of each pixel. It provides a measure of the biodiversity loss when vegetation is burned in the pixel *i*.

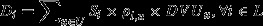
2

Finally, a normalized index *DNC*_*i*_ was calculated so that 
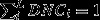
:

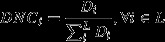
3

The second index is a vulnerability index (*Vul*), characterizing vegetation units' vulnerability to fire. It resulted from a combination of the vegetation units surface evolution (expansion or regression) computed from the potential (Table S1) and actual vegetation maps (*PS* and *AS*) and their time to recovery coefficient (*Time*). This coefficient was empirically organized into a hierarchy among vegetation units. Here, time to recovery coefficient is described as the delay needed for a certain vegetation type to recover from fire and normalized to set its maximum to 1. The vulnerability index was calculated for each vegetation unit separately and then attributed to each cell *i* according to the vegetation unit's composition (*ρ*_*i*,*u*_). This index denotes the capacity of an ecosystem to recover after a fire considering the major past dynamics. It provides a measure of the biodiversity disturbances when vegetation is burned in the pixel *i*.

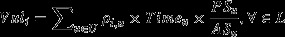
4

Finally, a global biodiversity loss index (*BL*) was obtained by combining the normalized diversity and vulnerability indices to account for both instantaneous loss (*DNC*) and long-term disturbances (*Vul*).

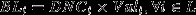
5

### Fire ignition probability modeling

The probabilities of fire ignition were estimated using *FINC* (Fire Ignition model in New Caledonia) (INC 2012). *FINC,* as a dynamic and spatially explicit model, is able to provide a geo-referenced fire ignition risk, over the mainland of New Caledonia, based on the physical environment such as the topography, climate, and some geographical indicators related to human influences such as population density and type of land property (See Appendix S1).

### Modeling fire behavior and severity

Fire spread modeling was implemented using *FLAMMAP* software (Finney 2005). The fires' spread and behaviors were simulated, taking into account spatially varying fuels, topography and weather (Hely et al., unpublished data) (See Appendix S1). FlamMap provided fire simulations which could not be evaluated with any field data. Consequently, fire hazard predictions need to be adjusted with the subsequent error made here and must remain probabilities. For every simulated fire ignited on a specific cell *j*, the fire line intensity *I*_*i,j*_ (kW/m) and fire spread rate *RoS*_*i,j*_ (m/min) were recorded in each cell *i* over the burnt area (denoted *B*_*j*_ in the following). To characterize fire severity, which is defined as the fire severity, that is, the magnitude of fire impact on wildland systems causing the loss or change in organic matter above ground and belowground (Keeley 2009), we calculated a severity index for each cell *i* potentially burned by a fire ignited on a specific cell *j* (*Sev*_*i,j*_) combining the fire line intensity (i.e., energy release, heat per length unit of fire front, *I*_*i,j*_) and the residence time (defined as 1/*RoS*_*i,j*_) (Whelan 1995) as follows:

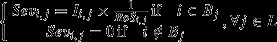
6

### One-off event fire impact on biodiversity loss and its associated risk

The expected fire impact of a one-off event (*Ed*) is a sum of the fire impact on all objects (*i.e*., affected cells). It provides a quantitative estimate of the damage caused by a fire ignited in a particular cell. Here, the concerns are about vegetation diversity, so fire impact on biodiversity loss was highlighted. Every fire ignition cell *j* of the mainland is associated with the resulting burnt area where fire behavior varied in terms of intensity and impact on the diversity. Biodiversity loss was calculated for all cells of the burnt summed over each burnt area and finally reported on the fire ignition cell *j* to determine the specific fire effects bound to that ignition cell.

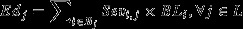
7

One-off event risk (*OeR*_*j*_) is defined as the product of the probability of a fire starting in a particular cell *j* (*FIP*_*j*_) and its expected impact (*Oe*_*j*_). This risk was normalized to values between 0 and 1. An average or daily risk could be calculated according to the ignition probability product used (daily or 10 years average).

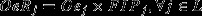
8

The output of a one-off event is of particular importance when evaluating the impact of an ignited fire and whether consequent emergency measures would need to be deployed.

### Burn probabilities and associated multi-event risk of impacts on biodiversity loss

Burn probability modeling simulates the ignition effects and spread of a large number of fires to calculate spatially explicit landscape level burn probabilities (Miller et al. 2008). In this study, the burn probability (*BP*) index was derived from the approach described in Finney (2005). It did not take into account land cover changes after being burned to simulate next fire spread and did not distinguish varying fire line intensities and spread rates within the burnt area of each independent fire. Indeed, taking into account such dynamics represents a very complex and computation time consuming process.

Based on the individual fire simulations computed using FLAMMAP (see section “Modeling fire behavior and severity”), the resulting burnt areas were recorded and compiled into a cumulative matrix of burnt area. The term *BAM*_*i,j*_ of the burnt area matrix (*BAM*) corresponds to the propagation of a fire ignited in a given 300 m × 300 m cell *j* and its impact on another cell *i* by:

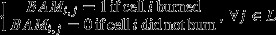
9

Originally, the burn probabilities index for the cell *j* is the ratio between the number of times the cell *i* burned (the row sum of *BAM*) and the total number of simulated individual fires. The Finney's method was improved to take into account a nonuniform fire's occurrence distribution based on the averaged fire ignition probability (*FIP*) to reach a more realistic burn probability pattern that included among other variables the human influences (see *Fire ignition probability modeling* section):

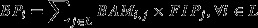
10

Multi-event risk (*MeR*_*i*_) was calculated by combining the updated burn probability (*BP*_*i*_) and the biodiversity loss index (*BL*_*i*_) to represent the likely impact of fire to burn a given pixel *i*.

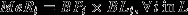
11

Multi-event risk was normalized to values between 0 and 1.

This output represents the likely biodiversity loss when vegetation faces recurrent fires. This metric allowed managing and preventing fires in the long-term perspective.

## Results

### Two indices to evaluate biodiversity loss under wildfire conditions

Diversity and vulnerability indices were calculated for each vegetation unit (Table1) and then attributed to each cell according to its composition in vegetation units.

Concerning the diversity contents index, the three raw ratios (*TS*,*ES,* and *SES)* represent different metrics to assess the part of biodiversity contained in each vegetation unit relatively to the total biodiversity observed all over the main land of New Caledonia. If the target is to organize the vegetation units into a hierarchy in terms of number of species, *TS* is a good indicator. If the objective is to focus on species confined to New Caledonia, ES and SES are more suitable. Nevertheless, the pairwise correlations of the ratios values (0.98, 0.95, and 0.94) indicate that the proportions of endemic species and specific endemic species do not vary significantly from a vegetation unit to another and that all these metrics are consistent in New Caledonia.

The diversity index values (*D*) represent an estimation of the part of biodiversity contained in the 300 × 300 meter pixel relatively to the entire main island's diversity. Three main levels of diversity could be distinguished with dense humid forest (DHF) on calcareous substrates and savannas (∼10^−5^), DHF on ultramafic and sedimentary-volcanic substrates and middle/low altitude maquis (∼10^−4^) and high altitude maquis, sclerophyll forest and mangrove (∼10^−3^). These model outputs suggest that a potential loss of one pixel (9 ha) of sclerophyll forest or mangrove is quantitatively equivalent to a loss of 100 pixels (900 ha) of savannas in terms of impacted number of species (total, endemic and specific endemic species).

The vulnerability index globally identified the primary units with the highest potential to be impacted by wildfires (*Vul* > 0.6), the mixed units (*Vul* ∈ [0.05, 0.08]) and the secondary units (null index). DHF on calcareous substrates appeared less vulnerable than other DHF because likely less exposed. Finally, maquis exhibit high species diversity (especially high altitude maquis) but are characterized by a relatively low vulnerability index (0.05 and 0.08 for middle/low and high altitude maquis, respectively) due to their composition of both primary and secondary vegetation.

**Figure 3:**
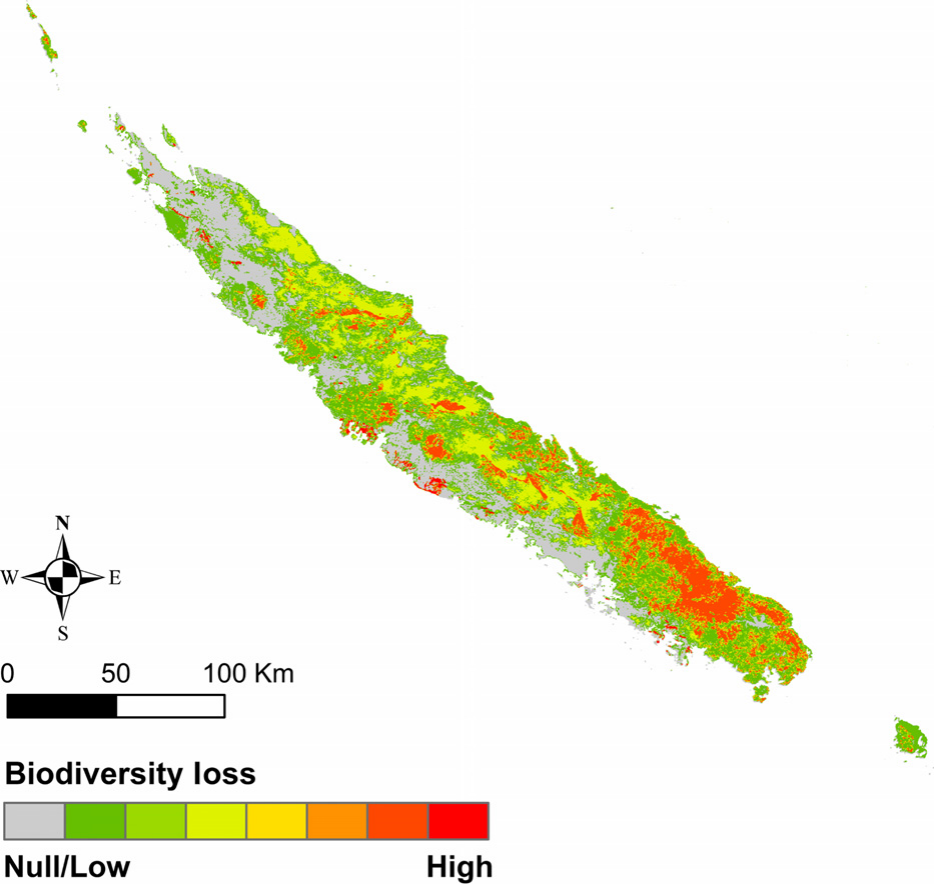
Potential biodiversity loss index mapping representing both direct potential loss through diversity and long-term potential loss through vulnerability. This index varied from 0 for secondary units (e.g., savannas) to 0.083 for sclerophyll forests.

The biodiversity loss (Fig.3) was selected as the index of interest to further evaluate wildfire impact: the higher the index, the more valuable the corresponding ecosystem in terms of biodiversity preservation. Four vegetation units have a high biodiversity loss index (Table1, Fig.3): sclerophyll with both a high vulnerability index and a high diversity, DHF on ultramafic substrates as the second most vulnerable and richest vegetation unit, DHF on sedimentary–volcanic substrates and high altitude maquis both having high biodiversity richness although less vulnerable. DHF on calcareous substrates and middle/low altitude maquis were identified with a moderate biodiversity loss index with moderate vulnerability indices either due to the low exposure to wildfire or to the mixed primary/secondary vegetation nature. Finally, savannas and secondary thickets have a nil potential biodiversity loss values due to its strictly secondary nature.

### Two different types of impact risks on biodiversity loss

**Figure 4:**
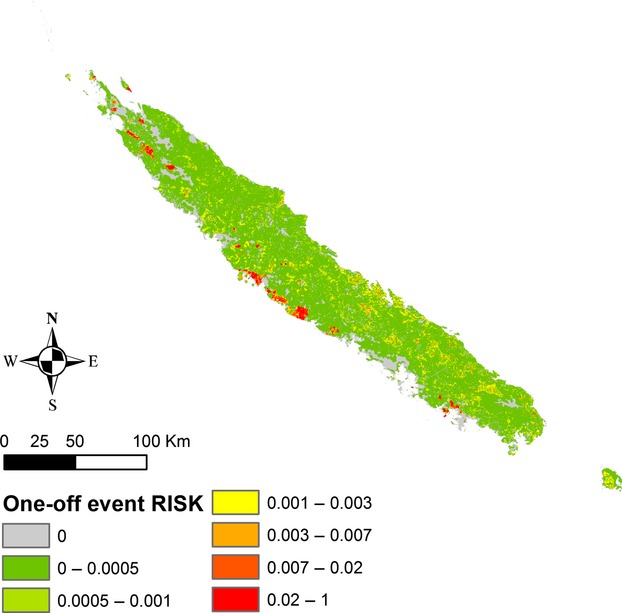
One-off risk associated to one-off impacts of fire on biodiversity loss combined with fire ignition probability. Normalized risk values appeared continuous and highly graduated with small areas displaying high risk value.

Fire impact from one-off fire ignition events was calculated for every ignition cell computing fire severity (magnitude) and biodiversity loss (Figure S4). The associated risk of a one-off event represented the likelihood of such an impact combined with the fire ignition probability (Fig.4). The expected impact map (see Figure S3) localized the areas where the ignition conditions were met and where a fire would likely spread.

**Figure 5:**
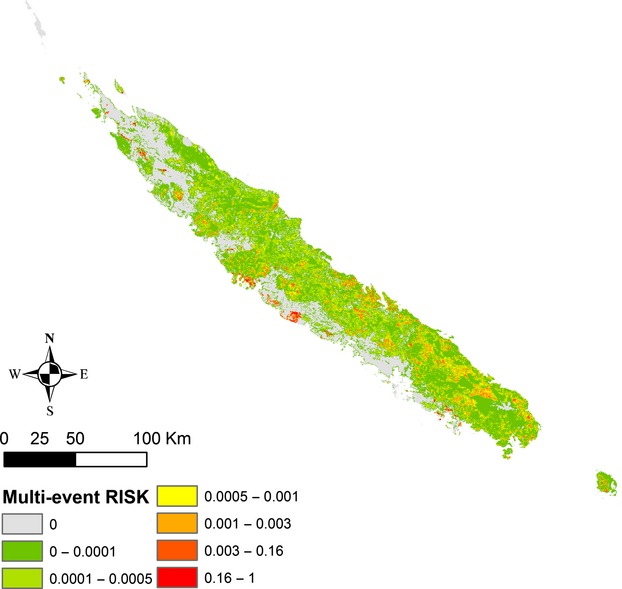
Multi-event risk associated to burn probability impacts of fire on biodiversity loss. Normalized risk values appeared continuous and highly graduated with small areas displaying high risk values.

Multi-event burn probabilities provided useful information about the fire spatial distribution patterns highlighting the most likely human and landscape-driven patterns (Figure S5). Multi-event risk was calculated by combining multi-event burn probabilities with the biodiversity loss index (Fig.5).

The corresponding high risk surface is smaller while considering the multi-event approach than while considering the event-based approach. Indeed, in the case of a one-off event risk, the values are based on the accumulated impact on a burnt area and include specific location of vulnerable biodiversity, as well as vegetation interface areas with low biodiversity richness but where a fire ignited in it can reach areas at risk.

**Figure 6:**
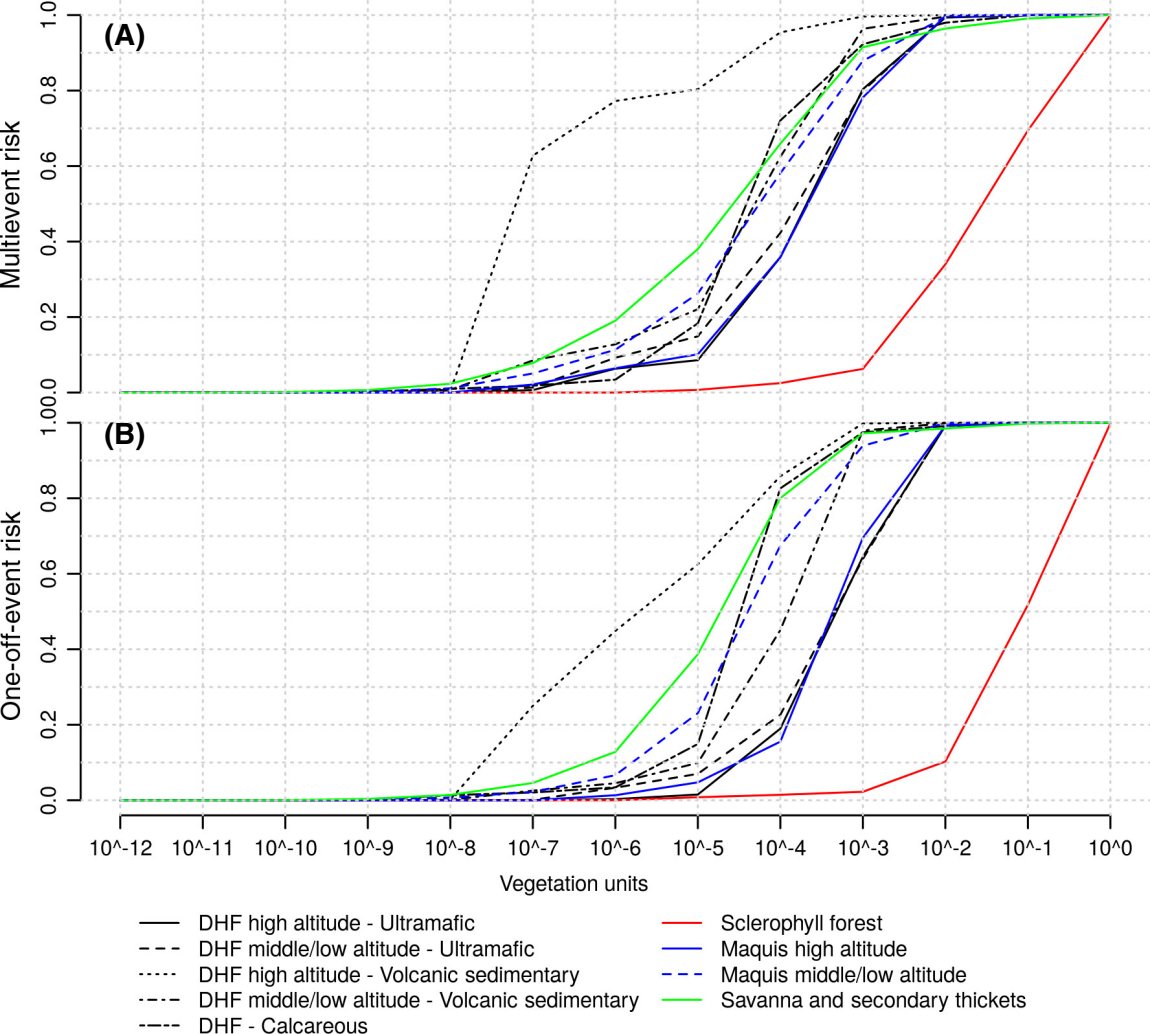
Cumulative distributions of the risks (multi-event (A) and one-off event (B)) across the whole main island of New Caledonia for each type of vegetation unit.

The cumulative distributions of both indices across the main vegetation units of New Caledonia can be found in Figure6A and B. It should be noted that the estimated risk takes a continuous range of values since percentage of vegetation units (*ρ* ∈ [0, 1]) was introduced in the risk calculation. Based on these distributions, we can observe that the risk decreases exponentially when all the conditions are not met for a significant impact on biodiversity (low potential biodiversity loss or low probability of ignition). Considering a multi-event risk above a threshold of 10^−1^, the principal threat on the biodiversity is concentrated within around 48% of the sclerophyll forest (see Fig.6A). If this threshold is set to 10^−2^, then 90% of this vegetation unit should be preserved from multiple fires to minimize the impact on biodiversity. For a 10^−3^ threshold, 98% of the sclerophyll forest, as well as 30% of the maquis (high altitude), 36% of DHF (high altitude – Ultramafic and Calcareous) should be monitored. Considering the one-off event risk (Fig.6B), for a to 10^−2^ threshold, a fire ignited within a surface related to 66% of the sclerophyll forest but also within 3.6% of savanna and secondary thickets and 2% of DHF (calcareous) could have a significant impact on the biodiversity.

### Analysis of distribution patterns in a restricted area

**Figure 7:**
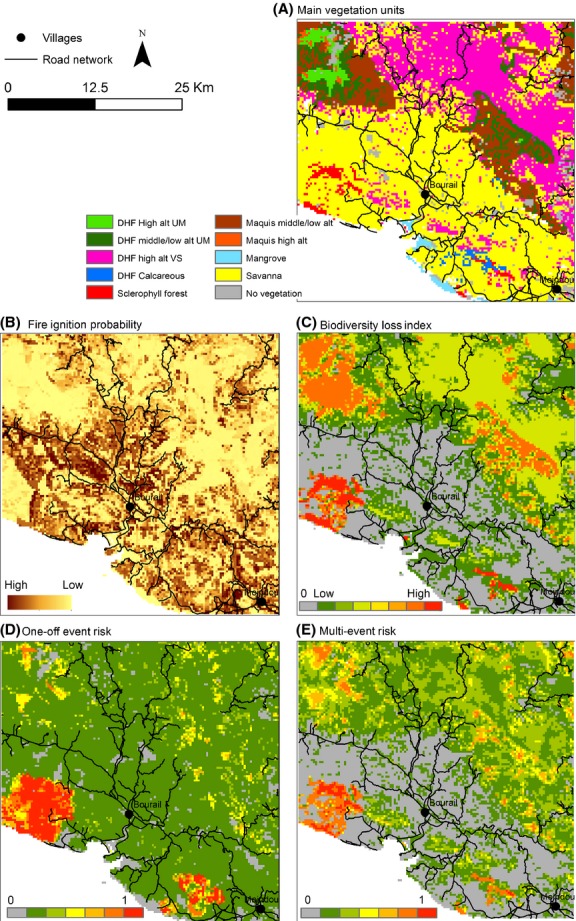
Study case around Bourail and Moindou villages on the west coast of New Caledonia mainland.

Around the villages of Bourail and Mouindou, the landscape is diverse enough to provide clues to understanding the risk patterns considering the main input characteristics (Fig.7). The diversity of cases described at the island scale can be highlighted in this study case. Firstly, it should be noted that the sclerophyll forest fragments and DHF on ultramafic substrates are clearly at risk in this area. Secondly, low fire ignition probabilities involve less risk, particularly in anthropogenic-free influence (far from villages or roads). Finally, vegetation unit interfaces, especially between secondary (or mixed) and primary units, displayed higher risk (considering equal *FIP*) which was taken into account by the accumulated impact on given burnt areas. For instance, fires ignited in maquis or savannas would impact the neighboring dense humid forest or sclerophyll forest areas.

## Discussion

In this study, we have presented a modeling approach to investigate the potential impact of wildfire on the biodiversity of the different types of vegetation to preserve the areas presenting highest fire risks because of their higher level of diversity, exposure, and vulnerability.

This information is important, recent changes in both climate (increased drought) and societal factors (more frequent fire ignitions) may transform traditional fire regimes (Ibanez et al. 2013b) and increase the negative effects upon vegetation, soil and human values. In addition, frequent and large wildfires, especially during El Niño years (Barbero and Moron 2011), may cause extensive damage in New Caledonia.

### Fire risk evaluation and limitation of the modeling approach

Impact of fires could be calculated using either a vulnerability measure related to the degree of exposure only (Chuvieco et al. 2010; Guettouche et al. 2011) or a quantitative measure of potential losses (Ager et al. 2007; Massada et al. 2009; Roloff et al. 2012). The biodiversity loss index computed here combined both direct potential loss through the diversity index and long-term potential loss through the vulnerability index. The main vulnerability components are exposure, sensitivity, and adaptive capacities as defined by the intergovernmental panel on climate change (http://www.ipcc.ch). Every vulnerability components were taken into account in our study in particular through the long-term surface evolution calculated for each vegetation units and the time to recover after a fire.

As all approaches based on modeling, this framework is a simplification of the reality. In particular, in this study, due to a lack of data, we chose to work at a vegetation unit level and the impacts on specific species are not taken into account. Also, we did not take into account the different susceptibility of vegetation units (or ecosystems) to fires. In the tropics, as most fires can be categorized as in surface fires, the bark thickness of bole has emerged as a good proxy of postfire tree top kill (e.g., Lawes et al. 2011; Brando et al. 2012)). As a result, ecosystems with thick bark such as savannas are less susceptible to fire than ecosystems with thin bark such as humid forest (Hoffmann et al. 2003; Lawes et al. 2011; Brando et al. 2012). In this way, a recent review by Wolfe et al.(2014) shows that at any given stem diameter, moist forests had a thinner bark than a dry forest which consecutively had a thinner bark than savannas. Another ecological characteristic which can be used to infer the susceptibility of vegetation units to fires is the resprouting capacity (i.e., the capacity to produce regrowth from dormant vegetative buds after fire damages, see Higgins et al. 2000; Gignoux et al. 2009). In most fire-prone ecosystems such as savannas or shrublands, species have a high a resprouting capacity, while it is low in ecosystems poorly adapted to fire such as moist forests. For instance in New Caledonia, Jaffré et al. (1997) has shown that most of the species from maquis resprout after fires and Ibanez et al. (2012) shows the same for the savanna dominant tree species (*Melaleuca quinquenervia*).

We consequently expect the biodiversity loss to be higher in dense humid forest than in sclerophyll shrubland and dry forest and in savannas. These relative proxies (bark thickness and resprouting capacity) could have been added to the vulnerability index (*Vul*_*i*_) calculation but would not have had any quantitative effect in here as it appeared proportional to the time to recovery coefficient for each vegetation unit.

Fire planning and risk assessment are concerned with how often fires burn (referring thus to burn probabilities) and what effect they would have on fire propagation values (fire effect associated to a given fire behavior). More precisely, the wildfires' impact would vary according to fire spatial patterns and the values of the impacted objects. This definition was given by Finney (2005) and was then used in several wildfire risk analyses (Ager et al. 2007; Massada et al. 2009; Roloff et al. 2012). On the other hand, an original fire risk scheme has been proposed by Chuvieco et al. (2010), taking into account specific fire behaviors and ecosystem vulnerability. Our study has provided for both approaches: a one-off event risk (similar to the Chuvieco risk assessment) and a multi-event risk representing a structural risk (similar to the Finney's risk assessment). While the former is usually computed to evaluate damages, the latter is an update to the quantitative risk assessment suggested by Finney (2005). Combining both event-driven and structural potential damages, provided a complete and integrated wildfire risk assessment for concerned valuables (here vegetal biodiversity).

In both the one-off event and multi-event risk assessments, the spatially distributed fire ignition probabilities (*FIP*) allowed not only a probabilistic component in our risk analysis but also provided a risk estimation that took into account daily weather forecast (provided by the French weather forecasting agency, Meteo France) and all landscape parameters as well as geographical and anthropogenic indicators (in particular population density and type of land property). Integrating human influences through the fire ignition probability into the wildfire risk analyses was essential to providing an adaptable and useful tool to concretely manage, minimize, and control wildfire impact in the New Caledonian context. Human and landscape-driven burn probabilities revealed a pattern where the highest values stand around human infrastructure such as roads (50% and 80% of the fires occurred within 500 m and 1 km of a roadway, respectively) and villages. They are also localized around the most flammable vegetation units, such as savannas and sclerophyll forest. The fact also that the vegetation interfaces between secondary and primary units displayed high risk suggests that those specific interfaces should represent priority zones for fire effects mitigation.

Most of the risk for biodiversity is located around these interfaces between fire-prone vegetation units and vegetation unit having a low tolerance (e.g., Ibanez et al. 2013a) to such savannas fire where the damages could be dramatic through understorey fire propagation (e.g., Carvalho et al. 2010).

### Urgent preservation measures needed

Currently, the only information available in New Caledonia, that aims at predicting and preventing the wildfire impact, is a weather prediction index (METEO France 2007) which has been integrated into this study through fire ignition probabilities. Although, wildfire's impact on the actual vegetation units spatial patterns have been recognized (Jaffré et al. 1995) extensive wildfires still occur with significant damage (e.g., at *Montagne des sources* 2005 and *Creek Pernod* 2013) to this biodiversity hotspot (Myers et al. 2000). Risk assessments need to be seen as a management decision-making tool by providing comprehensive risk analyses to ensure a wildfire control policy (Fairbrother and Turnley 2005).

As mentioned earlier, small localized areas displayed high fire risks values. These specific areas have to, urgently, be preserved in New Caledonia. Concretely, sclerophyll forests and adjacent regions appeared to be the most endangered and damageable areas in New Caledonia. Indeed, the patterns relating to the one-off event highest risk (*OeR* > 0.02) are all located around the remaining sclerophyll forest fragments and are representing no more than 211 km² of the mainland surface (i.e., 0.012%). Furthermore, according to a multi-event risk analysis, the sclerophyll forest and adjacent areas representing 30.24 km² (thus 0.0018% of the mainland area) appear to be highly exposed (*MeR*> 0.16). Maquis and areas adjacent to dense humid forest on ultramafic soils also appear endangered with *MeR* ∈ [0.001, 0.16] which corresponds to 1130 km² (0.067% of the mainland surface).

These scenarios may be considered as excessively pessimistic as we do not take into account the positive role of fire on certain specific species but the positive impact in terms of biodiversity is very low in New Caledonia because the vegetation units for which fire is a positive agent for ecosystem functioning such as savannah have a very low biodiversity index. In other contexts, this positive role should be taken into consideration.

### Further applications for New Caledonia's fire management

Specific risk assessments for each individual potential fire ignition and the subsequent potential damage within the associated burnt area appear to be an indispensable tool in New Caledonian context. Indeed, around 32% of the mainland is covered by savannas, anthropogenic, and fire-prone vegetation. In such a study, estimating wildfire risks on biodiversity needs to consider habitat features as well as fuel quantity (Haslem et al. 2011). Therefore, habitats with a high biodiversity loss index in the New Caledonian landscape and specifically the edges between habitats have to be monitored.

The impact of a one-off event provided an evaluation of all impacts a fire could have if ignited at a specific location. One-off event associated risk allowed the localization of the most likely ignition areas and with potentially extensive damage. Typically, fires ignited within savannah regions, situated at the edge of dense humid forest(Ibanez et al. 2012, 2013c) and/or maquis could cause extensive damage to biodiversity and would have to be controlled with careful monitoring. Emergency actions could aim to limit a specific fire spread (lessen burn severity or intensity) those known to have high impacts or focus on targeting high risk areas to limit one-off fire ignitions.

Spatially explicit information on the burning probability is necessary for virtually all strategic fires and fuel management planning, optimizing fuel treatments, and prevention activities. The use of burn probabilities to manage ground planning for long-term fire prevention already been assessed (Miller et al. 2008). Long-term planning actions would be employed based on the burn probability and multi-event risk maps. Different actions could be engaged to minimize fire ignitions, in hazardous regions, through fuel treatment or public awareness (Chuvieco et al. 2004; Fernandes 2009) or to reduce the expected fire effect by lowering fuel loads (Fernandes and Botelho 2003).

## Operability and Perspectives

This model has provided useful tools to concretely manage fire impact. Daily outcomes (risk assessment, impact and fire ignition probabilities) are available on line at http://deployeur.univ-nc.nc/inc/modele.html for the fire and biodiversity service stakeholders. Other analyses, that take into account fire department logistic resources, would have to be carried out to provide specific thresholds to the relevant New Caledonian governmental institutions and online daily risk assessments to improve the tool's operability.

Nevertheless, this study provides only information at a country scale that could be improved particularly by distinguishing intermediate units from maquis to forest, as tall maquis, trees-savannas to herbaceous savannas, or sclerophyll forest to dense humid forest (mesic forest) or intact DHF to damaged DHF. Further research works may also be conducted to improve the localization of the biodiversity (the micro-endemism in particular is not considered in this study) and to deepen the concept of resilience for each vegetation unit. Considering species distributions, if this information is available, may also make the estimation of wildfire impacts on biodiversity more accurate asthe response to fire is species specific and that the vegetation unit composition can vary from one location to another. Finally, assessing other ecological stakes such as the ecosystem services and estimating indirect fire threat such as the fragmentation of habitats constitute concrete research directions that may be of interest for biodiversity preservation.

## Supporting Information

**Appendix S1.**List of supporting tables and figures. **Table S1.** Primary vegetation unit environmental characteristics according to (Jaffré and Veillon). **Table S2.** Confusion matrix for 6 classes to validate vegetation unit mapping (predicted data) based on georeferenced observation data points. Values are in percent. **Table S3.** Overall prediction accuracy value according to an error distance revealed a maximum accuracy at 300 m. **Figure S1.** Primary (or climacic) main vegetation units potential distribution in New Caledonia according to environmental characteristics described in Table1. **Figure S2.** Comparison of inventorying distribution over 174 4 ares-quadrats and generalized values published for dense humid forest endemic and specific endemic species. **Figure S3.** Expected impacts of specific one-off fire events (referred to event-driven fires) in New Caledonia, calculated combining fire severity and biodiversity loss over the burned area and reported on the given pixel of ignition. **Figure S4.** Burn probability calculated combining every potential fire occurrences across New Caledonia (and their consequent burned area) with the given probability of fire ignition.

## References

[b1] Ager AA, Finney MA, Kerns BK, Maffei H (2007). Modeling wildfire risk to northern spotted owl (Strix occidentalis caurina) habitat in Central Oregon, USA. For. Ecol. Manage.

[b2] Bachmann A, Allgower B (2001). A consistent wildland fire risk terminology is needed. Fire Manage. Today.

[b3] Balch JK, Nepstad DC, Curran LM, Brando PM, Portela O, Guilherme P (2011). Size, species, and fire behavior predict tree and liana mortality from experimental burns in the Brazilian Amazon. For. Ecol. Manage.

[b4] Barbero R, Moron V (2011). Seasonal to decadal modulation of the impact of El Nino-Southern Oscillation on New Caledonia (SW Pacific) rainfall (1950–2010). J. Geophys. Res. Atmos.

[b5] Barlow J, Peres CA (2008). Fire-mediated dieback and compositional cascade in an Amazonian forest. Philos. Trans. R. Soc. B Biol. Sci.

[b6] Bowman DMJS, Balch JK, Artaxo P, Bond WJ, Carlson JM, Cochrane MA (2009). Fire in the earth system. Science.

[b7] Brando PM, Nepstad DC, Balch JK, Bolker B, Christman MC, Coe M (2012). Fire-induced tree mortality in a neotropical forest: the roles of bark traits, tree size, wood density and fire behavior. Glob. Change Biol.

[b8] Carvalho JA, Veras CAG, Alvarado EC, Sandberg DV, Leite SJ, Gielow R (2010). Understorey fire propagation and tree mortality on adjacent areas to an Amazonian deforestation fire. Int. J. Wildl. Fire.

[b9] Chuvieco E, Cocero D, Riano D, Martin P, Martinez-Vega J, de la Riva J (2004). Combining NDVI and surface temperature for the estimation of live fuel moisture content in forest fire danger rating. Remote Sens. Environ.

[b10] Chuvieco E, Gonzalez I, Verdu F, Aguado I, Yebra M (2009). Prediction of fire occurrence from live fuel moisture content measurements in a Mediterranean ecosystem. Int. J. Wildl. Fire.

[b11] Chuvieco E, Aguado I, Yebra M, Nieto H, Salas J, Martin MP (2010). Development of a framework for fire risk assessment using remote sensing and geographic information system technologies. Ecol. Model.

[b12] Cochrane MA (2001). Synergistic interactions between habitat fragmentation and fire in evergreen tropical forests. Conserv. Biol.

[b13] Cochrane MA (2003). Fire science for rainforests. Nature.

[b14] Dìaz-Delgado R, Lloret F, Pons X (2004). Spatial patterns of fire occurrence in Catalonia, NE, Spain. Landscape Ecol.

[b16] Fairbrother A, Turnley JG (2005). Predicting risks of uncharacteristic wildfires: application of the risk assessment process. For. Ecol. Manage.

[b17] Fernandes PM (2009). Combining forest structure data and fuel modelling to classify fire hazard in Portugal. Ann. For. Sci.

[b18] Fernandes PM, Botelho HS (2003). A review of prescribed burning effectiveness in fire hazard reduction. Int. J. Wildl. Fire.

[b20] Finney MA (2005). The challenge of quantitative risk analysis for wildland fire. For. Ecol. Manage.

[b21] Giglio L, Loboda T, Roy DP, Quayle B, Justice CO (2009). An active-fire based burned area mapping algorithm for the MODIS sensor. Remote Sens. Environ.

[b22] Gignoux J, Lahoreau G, Julliard R, Barot S (2009). Establishment and early persistence of tree seedlings in an annually burned savanna. J. Ecol.

[b23] Granzow-de la Cerda I, Lloret F, Ruiz JE, Vandermeer JH (2012). Tree mortality following ENSO-associated fires and drought in lowland rain forests of Eastern Nicaragua. For. Ecol. Manage.

[b24] Guettouche MS, Derias A, Boutiba M, Bounif MA, Guendouz A, Boudella A (2011). A fire risk modelling and spatialization by GIS. J. Geogr. Info. Syst.

[b25] Hardy CC (2005). Wildland fire hazard and risk: problems, definitions, and context. For. Ecol. Manage.

[b26] Haslem A, Kelly LT, Nimmo DG, Watson SJ, Kenny SA, Taylor RS (2011). Habitat or fuel? Implications of long-term, post-fire dynamics for the development of key resources for fauna and fire. J. Appl. Ecol.

[b27] Hayes JJ, Robeson SM (2011). Relationships between fire severity and post-fire landscape pattern following a large mixed-severity fire in the Valle Vidal, New Mexico, USA. For. Ecol. Manage.

[b28] Higgins SI, Bond WJ, Trollope WSW (2000). Fire, resprouting and variability: a recipe for grass-tree coexistence in savanna. J. Ecol.

[b29] Hochberg ME, Menaut J-C, Gignoux J (1994). The influences of tree biology and fire in the spatial structure of the West African Savannah. J. Ecol.

[b30] Hoffmann WA, Orthen B, Do Nascimento PKV (2003). Comparative fire ecology of tropical savanna and forest trees. Funct. Ecol.

[b31] Ibanez T, Borgniet L, Mangeas M, Gaucherel C, Géraux H, Christelle H (2012). Rainforest and savanna landscape dynamics in New-Caledonia: towards a mosaic of stable rainforest and savanna states. Austral Ecol.

[b32] Ibanez T, Curt T, Hely C (2013a). Low tolerance of New Caledonian secondary forest species to savanna fires. J. Veg. Sci.

[b33] Ibanez T, Hely C, Gaucherel C (2013b). Sharp transitions in microclimatic conditions between savanna and forest in New Caledonia: insights into the vulnerability of forest edges to fire. Austral Ecol.

[b34] Ibanez T, Munzinger J, Gaucherel C, Curt T, Hély C (2013c). Austral J. Bot.

[b35] INC (2012).

[b36] Jaffré T, Veillon J-M (1994).

[b37] Jaffré T, Veillon J-M, Rigault F, Dagostini G (1995).

[b38] Jaffré T, Veillon J-M, Pintaud J-C (1997).

[b39] Jaffré T, Bouchet P, Veillon J-M (1998a). Threatened plants of New Caledonia: is the system of protected areas adequate?. Biodivers. Conserv.

[b40] Jaffré T, Rigault F, Dagostini G (1998b). Impact des feux de brousse sur les maquis ligno-herbacés des roches ultramafiques de Nouvelle-Calédonie. Adansonia.

[b41] Jaffré T, Rigault F, Dagostini G, Tinel-Flambart J, Wulf A, Munzinger J (2009).

[b42] Kass GS, Shaw RF, Tew T, Macdonald DW (2011). Securing the future of the natural environment: using scenarios to anticipate challenges to biodiversity, landscapes and public engagement with nature. J. Appl. Ecol.

[b43] Keeley JE (2002). Fire management of California shrubland landscapes. Environ. Manage.

[b44] Keeley JE (2009). Fire intensity, fire severity and burn severity: a brief review and suggested usage. Int. J. Wildl. Fire.

[b45] Kitzberger T, Araoz E, Gowda JH, Mermoz M, Morales JM (2012). Decreases in fire spread probability with forest age promotes alternative community states, reduced resilience to climate variability and large fire regime shifts. Ecosystems.

[b46] Lawes MJ, Richards A, Dathe J, Midgley JJ (2011). Bark thickness determines fire resistance of selected tree species from fire-prone tropical savanna in north Australia. Plant Ecol.

[b47] Massada AB, Radeloff VC, Stewart SI, Hawbaker TJ (2009). Wildfire risk in the wildland-urban interface: a simulation study in northwestern Wisconsin. For. Ecol. Manage.

[b48] McCoy S, Jaffre T, Rigault F, Ash JE (1999). Fire and succession in the ultramafic maquis of New Caledonia. J. Biogeogr.

[b49] METEO France (2007). Atlas climatique de la Nouvelle Calédonie.

[b50] Miller C, Parisien M-A, Ager AA, Finney MA (2008). Evaluating spatially-explicit burn probabilities for strategic fire management planning. Model. Monit. Manage. For. Fires.

[b51] Mittermeier RA, Robles GP, Hoffman M, Pil-Grim J, Brooks T, Mittermeier CG (2004).

[b52] Morat P, Jaffre T, Tronchet F, Munzinger J, Pillon Y, Veillon JM (2012). The taxonomic reference base Florical and characteristics of the native vascular flora of New Caledonia. Adansonia.

[b53] Myers N, Mittermeier RA, Mittermeier CG, da Fonseca GA, Kent J (2000). Biodiversity hotspots for conservation priorities. Nature.

[b54] Nepstad DC, Verissimo A, Alencar A, Nobre C, Lima E, Lefebvre P (1999). Large-scale impoverishment of Amazonian forests by logging and fire. Nature.

[b55] Perry GLW, Sparrow AD, Owens IF (1999). A GIS-supported model for the simulation of the spatial structure of wildland fire, Cass Basin, New Zealand. J. Appl. Ecol.

[b56] Pinol J, Castellnou M, Beven KJ (2007). Conditioning uncertainty in ecological models: assessing the impact of fire management strategies. Ecol. Model.

[b57] Roloff GJ, Mealey SP, Bailey JD (2012). Comparative hazard assessment for protected species in a fire-prone landscape. For. Ecol. Manage.

[b58] Siegert F, Ruecker G, Hinrichs A, Hoffmann AA (2001). Increased damage from fires in logged forests during droughts caused by El Nino. Nature.

[b59] Stevenson J (2004). A late-Holocene record of human impact from the southwest coast of New Caledonia. Holocene.

[b60] Stott P (2000). Combustion in tropical biomass fires: a critical review. Prog. Phys. Geogr.

[b61] Syphard AD, Radeloff VC, Keuler NS, Taylor RS, Hawbaker TJ, Stewart SI (2008). Predicting spatial patterns of fire on a southern California landscape. Int. J. Wildl. Fire.

[b62] Trabaud L (1994). Post-fire plant community dynamics in the Mediterranean Basin. The role of fire in Mediterranean-type ecosystems.

[b63] Whelan RJ (1995). The ecology of fire.

[b64] Williams RJ, Griffiths AD, RA Bradstock JW, Gill AM, Allan G (2002). Fire regimes and biodiversity in the wet-dry tropical savanna landscapes of northern Australia. Flammable Australia: the fire regimes and biodiversity of a continent.

[b65] Wolfe BT, Diaz GES, Van Bloem SJ (2014). Fire resistance in a Caribbean dry forest: inferences from the allometry of bark thickness. J. Trop. Ecol.

