# Supplementary material for: Wildfire risk for main vegetation units in a biodiversity hotspot: modeling approach in New Caledonia, South Pacific
Source: Ecol Evol. 2014 Dec 28;5(2):377–90. doi: 10.1002/ece3.1317 (PMC4314270; doi:10.1002/ece3.1317)
Supplement: Supplementary file 1 [file ece30005-0377-sd1.docx]

## Supporting Information

*Main vegetation units mapping*

Five main vegetation units are generally recognized in New Caledonia (Morat *et al.*, 2012): dense humid evergreen forest where average annual rainfall exceeds 1100-1200 mm), sclerophyll forest also referred as “dry forest” or “forêt sèche” (with average annual precipitation less than 1200mm and a marked dry season), mangrove, maquis, savanna (including herbaceous, woody and shrubby savannas) and secondary thickets. From those five main vegetation units, subclasses could be distinguished according to different substrates for dense humid forest and different elevation level for maquis. Actual cartography was based on the latest land cover data available derived from SPOT imageries computed in 2008 validated with a kappa coefficient of 0.755 (DTSI and Boyaud, 2008), elevation data (from Digital Elevation Model) and substrates (ValPedo, IRD). Eight vegetation units were distinguished integrating data from land cover, elevation and substrates. More precisely, we have identified three major dense humid forest types whether they grow on ultramafic, sedimentary-volcanic or calcareous soils, two maquis types according to the elevation level (low and middle altitude *versus* high altitude maquis), sclerophyll forest (also referred as dry forest), mangrove and savannas grouped with secondary thickets. Those eight actual vegetation units were mapped all over the main island of New Caledonia (Figure 2). Dense humid evergreen forest, sclerophyll forest and mangrove are the only real primary units, while maquis is a mixed unit naturally existing but also still expanding replacing dense humid forest on specific substrate after forest degradation.

To characterize the mapped vegetation units by specific attributes, we collected expert knowledge and data from the literature (Jaffré *et al.*, 1997;Jaffré *et al.*, 1998a;Jaffré *et al.*, 2009). For each vegetation type we assessed the richness (total number of species), endemism (species exclusively in New Caledonia) and specificity (endemism related to only one New Caledonia vegetation type). The actual and potential surfaces of each vegetation units (denoted *AS* and *PS* respectively) were also calculated based on corresponding maps (Figure1 and Supplementary Figure1). Potential surfaces were estimated through the cartography of putative primary vegetation unit spatial distributions in New Caledonia, based on expert knowledge for annual precipitations, elevation and soil nature (Supplementary Table 1). The potential distribution of primary vegetation units, assuming no anthropogenic impacts, has been mapped according to expert rules summarized in Supplementary Table1 (Supplementary Figure 2). Savannas and secondary thickets are strictly secondary units non-existent originally before human settlement and associated disturbance, that is why its potential area is null (Jaffré and Veillon, 1994).

However, in an anthropogenic and wildfire context, savannas have emerged and still increased despite of primary units.

Two validation levels were implemented to test the spatial accuracy of the cartography and to validate the use of general published attributes to characterize the main vegetation units. The first step of validation was implemented to validate the complete actual vegetation distribution map using accurate georeferenced data points of species specific of a vegetation unit. The given data points were selected through the intersection between two botanical databases (VIROT and FLORICAL databases (Morat *et al.*, 2012)). A confusion matrix was calculated between predicted (mapped) and observed vegetation unit (georeferenced database points) using a gradient distance buffer tolerance. The second validation step has been only done for dense humid forest (without any substrate or elevation distinction) for data availability reason. Actual distributions of endemic species and specific endemic species, as inventoried during field campaign among 4 quadrats (n=174), and generalized values (Jaffré 2009) were compared to insure the reliability of using generalized values to characterize vegetation units.

*Fire Ignition model*

The probabilities of fire ignition were estimated using *FINC* (Fire Ignition model in New Caledonia)(INC, 2012). *FINC* is a dynamic and spatially explicit model able to provide a geo-referenced fire ignition risk, over the mainland of New Caledonia, based on the physical environment such as the topography, climate, and some geographical indicators related to human influences. The model is dynamic and some input is updated at varying times: the vegetation growth and land use changes are updated every two months by computing the NDVI vegetation index whereas weather conditions are updated daily. *FINC* is grid based with a cell size set to 300m×300m and composed of three distinct modules that were combined within a Bayesian network to provide a geo-referenced global ignition risk. The parameters (*i.e.* the joint probabilities) were estimated by studying the fire ignition observations over 10 years. This model uses as input data: geographic data (land use, roads etc), daily meteorological data (fire weather index, precipitation etc) and physical parameters (slope, elevation etc). The performance analysis showed that this model is efficient with a kappa of 0.78.

*FLAMMAP Simulations*

Fire simulations were based on the minimum travel time algorithm (MTT) implemented in FLAMMAP3; that is a spatially explicit model that can efficiently simulate fire spread over complex landscapes assuming temporarily constant weather conditions (Finney, 2002). The assumption of constant weather conditions limits the feasibility of using MTT for simulating long fire events, but for shorter burn times, its results are acceptable (Finney, 2005). Moreover, it is impossible to obtain or simulate data for the limitless ignition combinations and weather conditions. Wildfire risk studies thus commonly focus on extreme weather conditions, since they favor the occurrence of large fire events that are harder to suppress and pose the highest risk (Finney, 2005). The methodology and the parameters were adapted to simulate putative extreme scenario of wildfire with extreme consequences by employing averaged extreme climatic conditions, the same high rate of spread for each vegetation units and a long propagation time.

Here, fire growth simulations were done for every 300m × 300m cell on the New Caledonian mainland map. The Loyalty Islands were not included in the whole wildfire impact and risk analysis because of the lack of fire/biodiversity issue in this region. As input: elevation, slope, aspect, fuel model, canopy cover, wind vectors (direction and intensity) and canopy characteristics (height, canopy bulk density, canopy base height and foliar moisture content) were provided. Specific fuel models were developed for that study by considering each vegetation unit separately and conducting litter controlled burning protocols (Hély, data not shown). Fuel models and wind vectors were determined according to weather conditions most favorable to fire propagation (*i.e.* extreme climatic conditions) in New Caledonia (METEO-France, 2007). Fires were simulated for eight hours, which corresponds with a whole afternoon of propagation. Such conditions are realistic in the New Caledonian environment which has low firefighting abilities, access difficulties, and night conditions favoring extinction. Burnt areas were therefore limited to a maximum of 441 cells (limitation due to the 8 hour propagation duration). The spatial resolution of calculations was fixed at 300m and the minimum travel paths interval to 300m.

Fire growth was simulated only for extreme climatic conditions, as suggesting by Finney (2005) in order to avoid a highly complicated algorithm development. Thus the most likely damaging scenario was considered in risk assessment analyses and particularly in fire spread simulations (fire spread time) which provided an evaluation of all the potential areas damaged.

### Vegetation units characterization and spatial distribution

The complete actual distribution map was validated at 60.8% of overall accuracy throughout the confusion matrix between mapped (predicted) and observed (databases) vegetation units. Introducing a distance error from 50 to 300m, overall accuracy was improved from 78% at 50m to 98.95% at 300m and kappa coefficient from 55% (at 50m) to 85.3% at 300m (Supplementary Table 3). Assuming a 300m error, which is corresponding to the spatial resolution of the whole fire risk model developed in this study, allowed to reach a very good accuracy level. Analyzing the spatial distribution of georeferenced data points used in that validation process showed that a significant proportion of points were misplaced at unit borders likely because of a resolution issue. Two classes recorded high commission errors (savannas and no vegetation) (Supplementary Table 2), while the main primary vegetation units displayed good validation scores.

Pertinence of using generalized values to describe the vegetation units in terms of number of species has been validated comparing endemic species and specific endemic species inventoried actual distributions with generalized values published (Supplementary Figure 2). In terms of endemic species, inventoried average value was higher (93% ± 5.6%) than the published value which can be partly explained by the differences in inventoried target species. Indeed, contrary to the field data, published value (82.1%) include all the species even those with low endemism rate such as epiphytic ones (for instance Orchidaceae). In terms of specific endemism, values matched well 57.62% *versus* 61% ± 19%.

Supplementary Table 1- Primary vegetation unit environmental characteristics according to (Jaffré and Veillon, 1994)

| ***Vegetation unit*** | ***Annual***  ***rainfall (mm)*** | ***Altitude (m)*** | ***Substrates*** |
| --- | --- | --- | --- |
| Dense humidforest |  |  |  |
| High altitude, ultramaficsubstrates | >1100 | 1000-1350 | Ultramafic |
| Middle/low altitude, ultramafic substrates | >1100 | <1000 | Ultramafic |
| High altitude, sedimendary& volcanicsubstrates | >1100 | 1000-1350 | Volcanic |
| Middle/low altitude, sedimendary& volcanicsubstrates | >1100 | <1000 | Volcanic |
| Calcareoussubstrates | >1100 | - | Calcareous |
| High altitude maquis | - | >1350 | Ultramafic |
| Middle/low altitude Maquis | <1100 | <1000 | Ultramafic |
| Sclerophyllforest  Mangrove | <1100  - | -  - | Volcanic  Humid |

SupplementayTable 2 – Confusion matrix for 6 classes to validate vegetation unit mapping (predicted data) based on georeferenced observation data points. Values are in percent.

|  | | **Observed data** | | | | | |
| --- | --- | --- | --- | --- | --- | --- | --- |
| **Predicted data** |  | DHF | Sclerophyll forest | Mangrove | Maquis | No vegetation | Savanna |
|  | DHF | **79.1** | 35.7 | 50.0 | 41.8 | 50.7 | 74.2 |
|  | Sclerophyll forest | 0.3 | **42.9** | 0.0 | 0.7 | 7.5 | 3.2 |
|  | Mangrove | 0.9 | 0.0 | **0.0** | 0.7 | 1.5 | 0.8 |
|  | Maquis | 16.2 | 21.4 | 0.0 | **49.7** | 33.6 | 15.3 |
|  | No vegetation. | 2.3 | 0.0 | 0.0 | 6.8 | **6.0** | 2.4 |
|  | Savanna | 1.3 | 0.0 | 50.0 | 0.4 | 0.7 | **4.0** |

Supplementary Table 3 – Overall prediction accuracy value according to an error distance revealed a maximum accuracy at 300m

| Error distance (m) | 0 | 50 | 100 | 150 | 300 |
| --- | --- | --- | --- | --- | --- |
| **Overall accuracy** | 0.608 | 0.780 | 0.8453 | 0.883 | 0.9295 |
| **Kappa coefficient** | 0.264 | 0.550 | 0.679 | 0.756 | 0.853 |


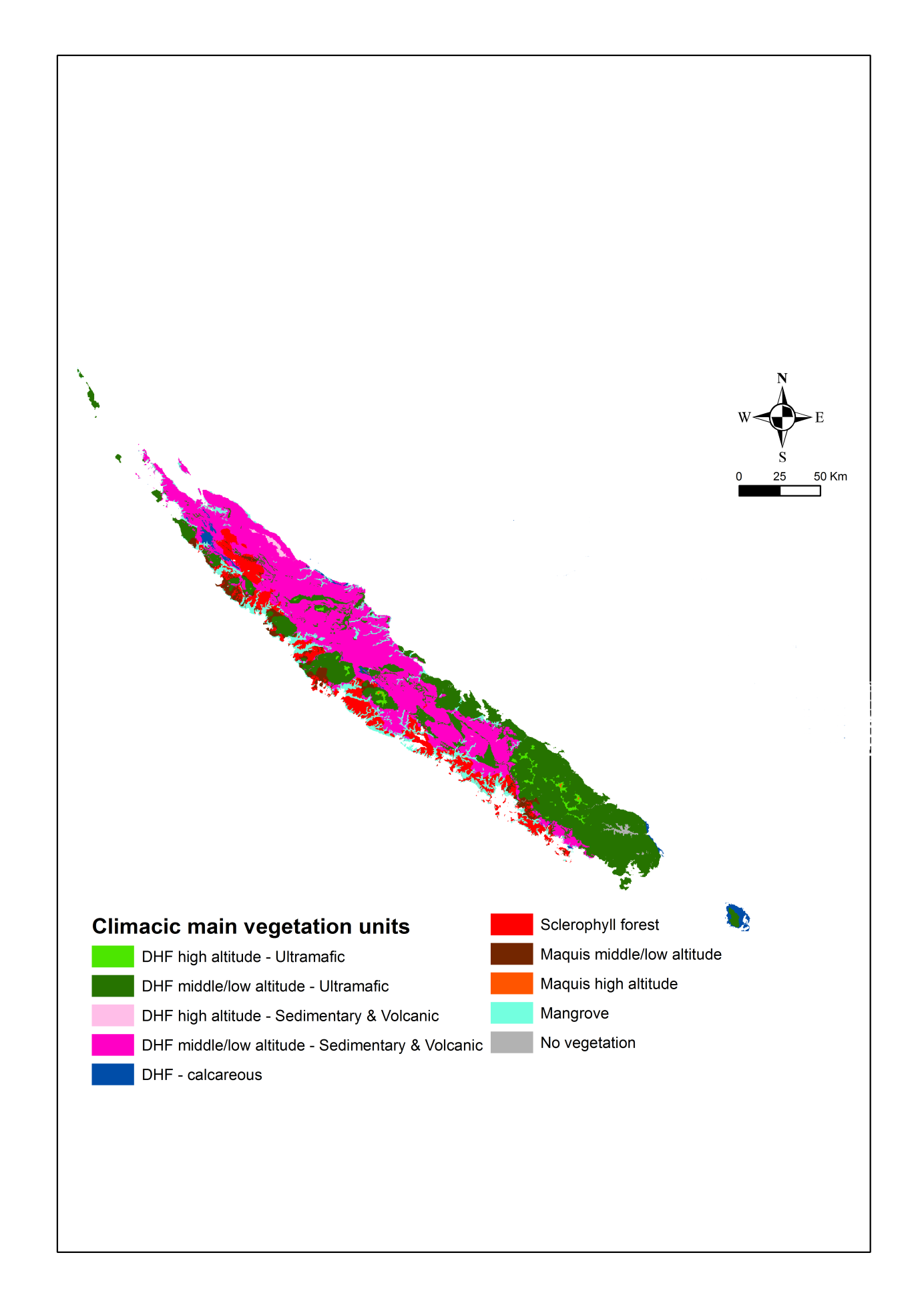


Supplementary Figure 4 – Primary (or climacic) main vegetation units potential distribution in New Caledonia according to environmental characteristics described in Table 1


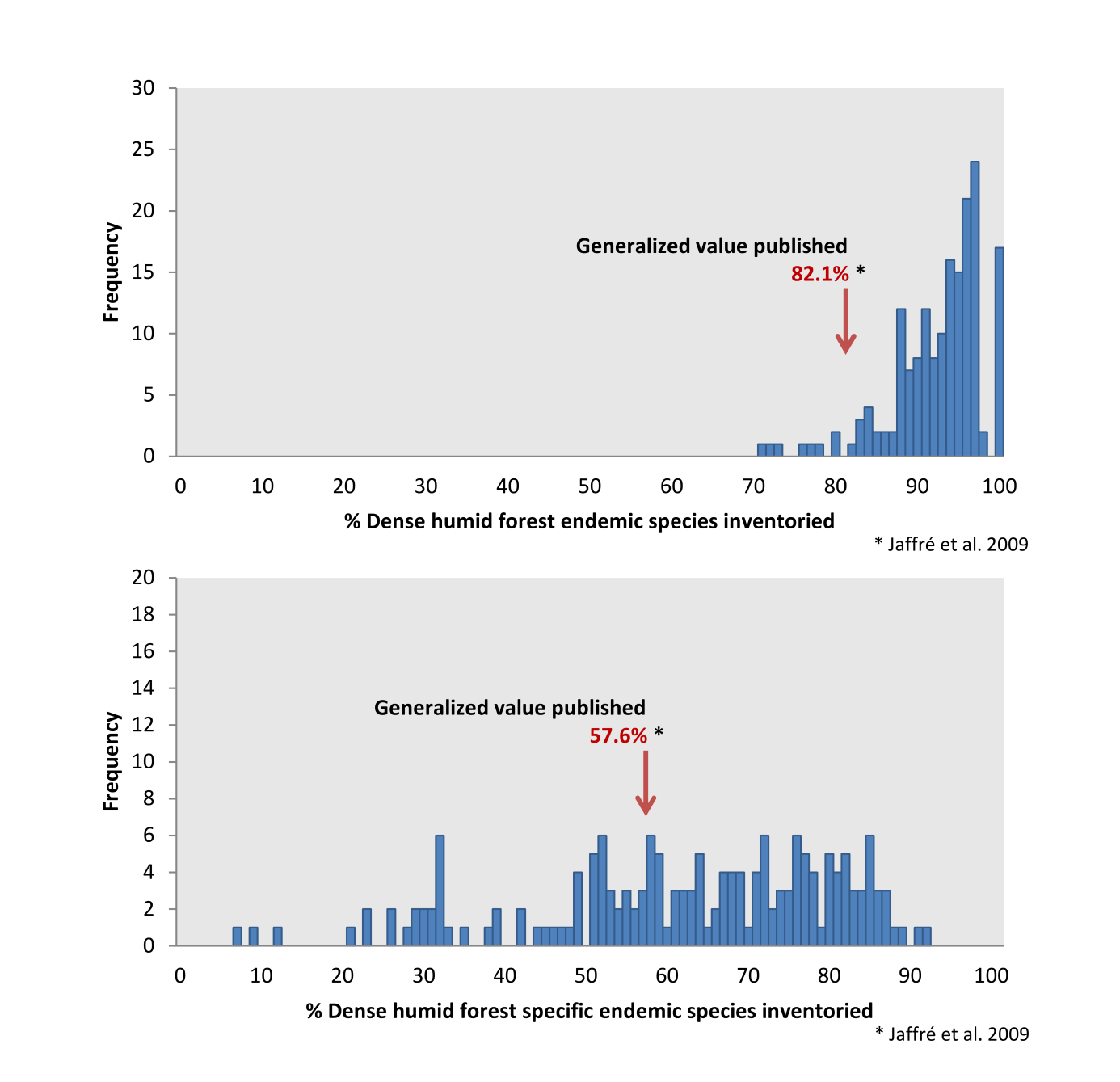
Supplementary Figure5 – Comparison of inventorying distribution over 174 4 ares-quadrats and generalized values published for dense humid forest endemic and specific endemic species.


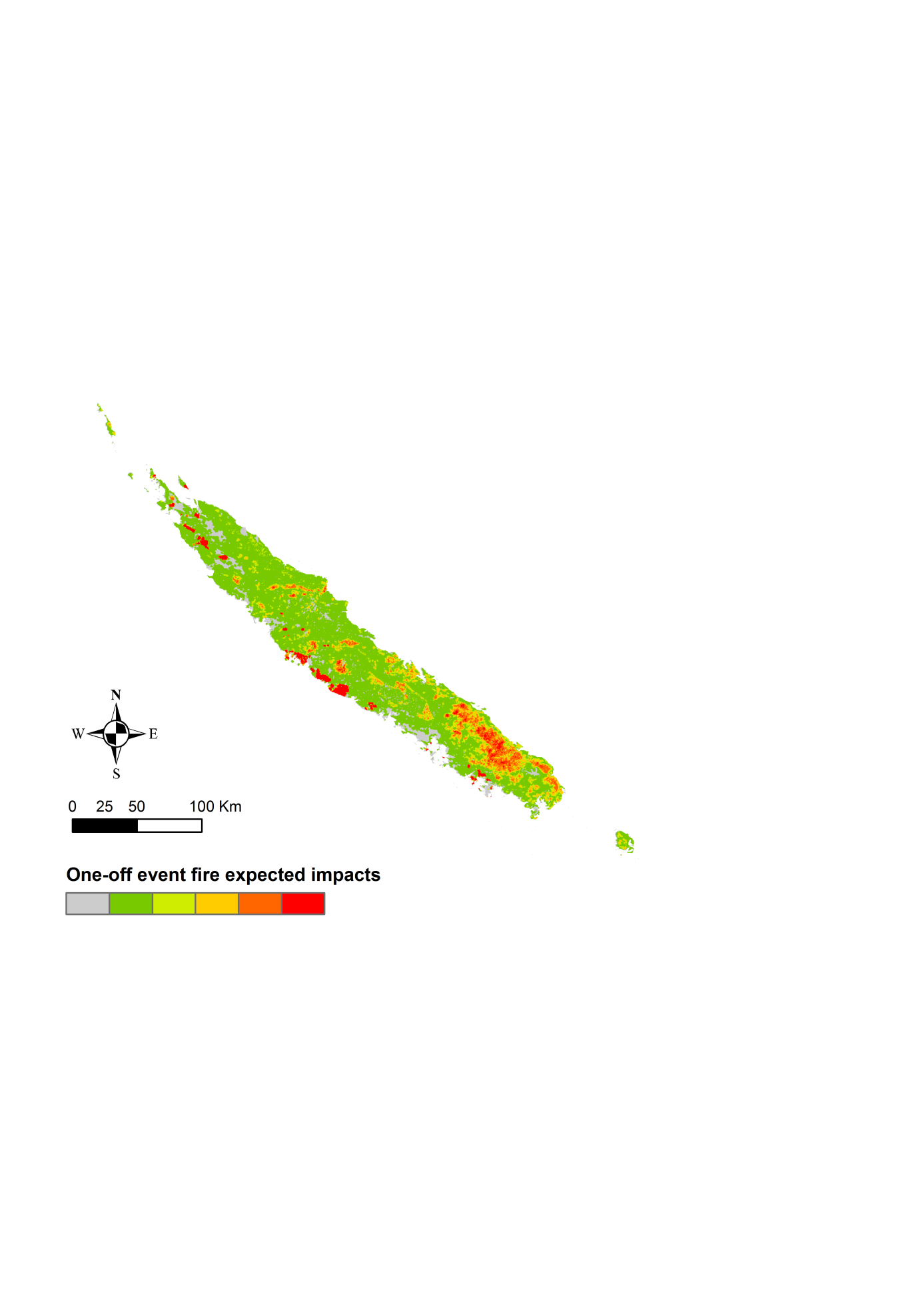


Supplementary Figure 6 – Expected impacts of specific one-off fire events (referred to event-driven fires) in New Caledonia, calculated combining fire severity and biodiversity loss over the burned area and reported on the given pixel of ignition


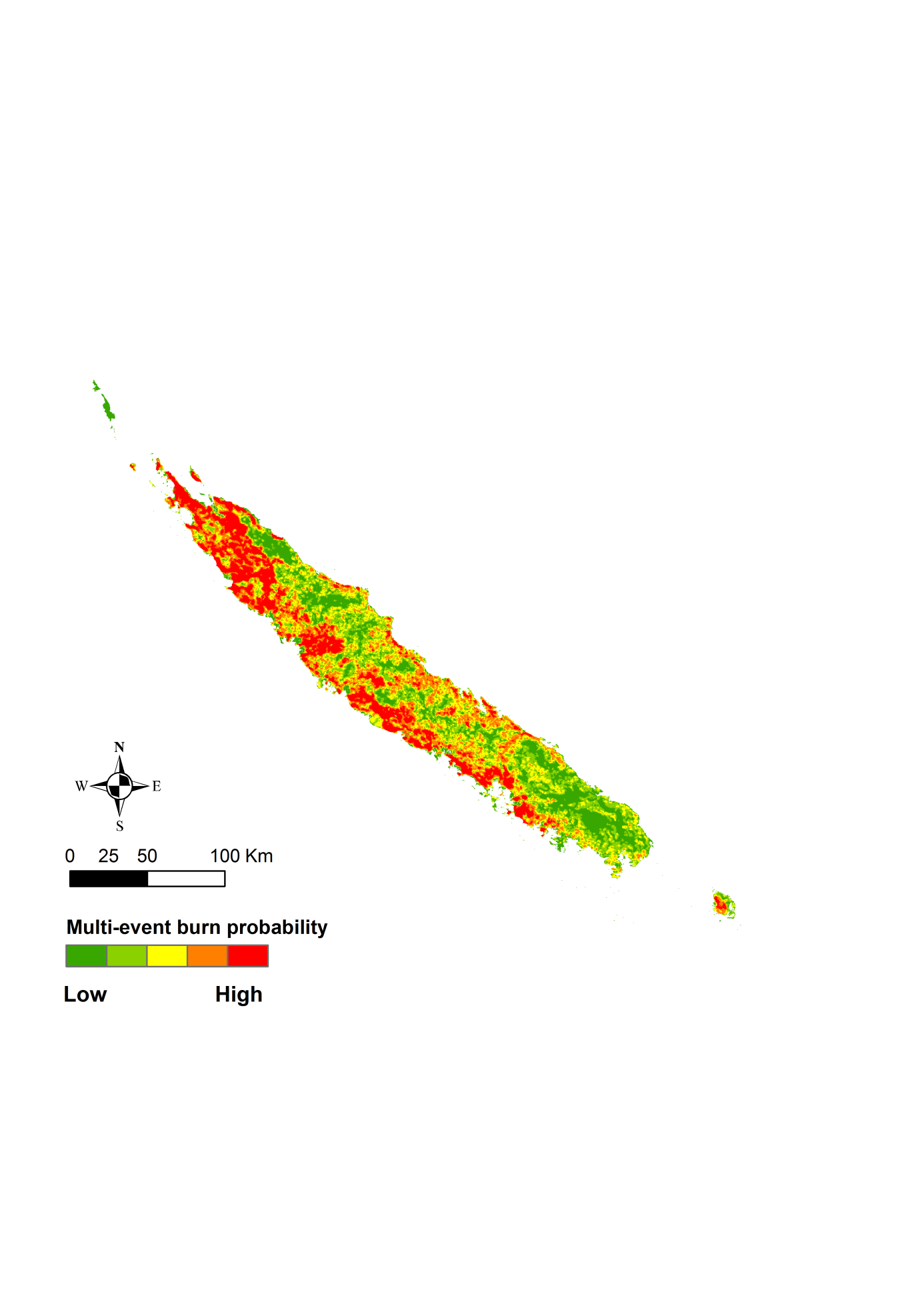


Supplementary Figure 4 –Burn probability calculated combining every potential fire occurrences across New Caledonia (and their consequent burned area) with the given probability of fire ignition. This specific multi-event burn probability appeared non-uniform as it took into account only the more likely fire occurrences according to the fire ignition probabilities which account for human parameters.
